# Utilization of Pine and Birch Juvenile Wood for Low-Density Particleboard Production

**DOI:** 10.3390/ma18051140

**Published:** 2025-03-04

**Authors:** Rafał Czarnecki, Dorota Dukarska, Jakub Kawalerczyk, Arkadiusz Filipski

**Affiliations:** Department of Mechanical Wood Technology, Faculty of Forestry and Wood Technology, Poznań University of Life Sciences, Wojska Polskiego 38/42, 60-627 Poznań, Poland; jakub.kawalerczyk@up.poznan.pl (J.K.); filipski-arek@wp.pl (A.F.)

**Keywords:** juvenile wood, wood-based materials, efficiency of wood resources, property of boards, strength, dimensional stability

## Abstract

This study investigated the effect of using juvenile pine and birch wood for the production of particleboards with lowered density, glued with urea-formaldehyde (UF) resin. The wood used was characterized by a number of annual rings ranging from 5 to 13, which ensured that only juvenile wood was used in the study. In addition to the basic characteristics of the wood particles obtained from this type of raw material, the density profiles of the manufactured particleboards, the internal bond, bending strength, modulus of elasticity, swelling, and water absorption after short-term water exposure (2 h) were also investigated. The results were compared to particleboards made from industrial wood particles from mature wood. It was found that particleboards made from juvenile pine wood exhibited higher internal bond than those made from juvenile birch wood. The bending strength of boards made from both types of juvenile wood was comparable to that of industrial particleboards; however, the modulus of elasticity of the particleboards made from juvenile pine was lower, which indicates reduced stiffness. These particleboards also showed higher swelling and water absorption, which may limit their durability under humid conditions. In contrast, birch boards exhibited lower internal bond, but their bending strength and modulus of elasticity were similar to those of industrial particles-based particleboards. Birch boards also showed slightly better water resistance than pine particleboards made from juvenile wood. However, their swelling remained higher than that of industrial particleboards. Overall, particleboards made from juvenile wood, especially birch, show good potential for further research.

## 1. Introduction

Particleboard is a widely recognized composite material manufactured by bonding wood particles with a synthetic resin or other suitable binder under the heat and pressure. It is valued for its cost-effectiveness, availability, versatility, and extensive applications in construction and furniture manufacturing [1,2]. The global annual production capacity of particleboard currently exceeds 100 million m^3^ and is expected to reach 122 million m^3^ by 2029 [3]. Asia leads as the largest producer of particleboard, followed by Europe, the Americas, Africa, and Oceania. Within Europe, countries such as Germany, Poland, Italy, Austria, and France rank among the top producers having a significant contribution to the global particleboard industry [4]. According to Benthien et al. [5], lightweight particleboards have emerged as an important area of research and development within the wood-based materials industry. As a result, many manufacturers of wood-based panels now offer lower density options as part of their product range. Low-density particleboards provide several advantages over traditional high-density boards, including reduced weight, improved thermal insulation, and enhanced machinability, making them easier to handle and process [6,7,8]. Moreover, they contribute to cost-effectiveness by requiring less raw materials for the production [9,10].

Reducing demand for wood is crucial because the wood-based materials industry is currently facing significant challenges due to increasing wood shortages, driven by factors such as over-exploitation of forests, climate change, and the growing demand for wood products across various sectors [11,12,13]. These shortages have put pressure on manufacturers and scientists to look for alternative sources of raw materials suitable for the production of particleboards. Examples of alternatives to commonly used softwood for the production of lignocellulosic particleboard include: agricultural residues [14,15], forest biomass [16,17], food industry by-products [18,19], fast-growing species [20,21], decayed wood [22,23], recycled wood [24,25] and fibrous materials [26,27]. Moreover, juvenile wood, which according to Shi et al. [28], is not the most suitable material for producing certain wood-based boards such as timber-based boards or plywood, is also being investigated as an alternative to mature wood for many years.

Juvenile wood, produced during the early stages of a tree’s development, typically consists of several annual rings located in the central portion of the cross-section. The exact number of these rings varies depending on the species and the research methods applied [29,30]. In comparison to mature wood, juvenile wood is characterized by higher lignin and lower cellulose content, lower density, higher microfibril angle, higher spiral grain angle, shorter cells with smaller diameter and thinner cell walls, lower modulus of elasticity and strength and higher longitudinal shrinkage [31]. Consequently, studies have shown that the use of juvenile wood may also affect the properties of wood-based panels. A study performed by Cloutier et al. [32], investigated the impact of juvenile wood proportion on the properties of OSB. The results indicate that the use of juvenile wood negatively affected thickness swelling, internal bond, and modulus of elasticity of resultant materials. The outcomes of another study confirmed that the increased proportion of juvenile wood negatively affected thickness swelling, internal bond, and modulus of elasticity of OSB [33]. On the other hand, the study investigating the potential of juvenile wood from nine underutilized wood species as a substitute for Norway spruce in OSB production found that juvenile wood from species such as larch, poplar, willow, and alder can be used to produce OSBs with mechanical properties comparable to those of spruce, thereby making them viable alternatives in the wood-based panel industry [34]. Similar findings were reported by Pugel et al. [35,36], who demonstrated that particleboards made from southern pine juvenile wood exhibited internal bond strength, modulus of rupture, modulus of elasticity, and durability after aging test comparable to those of reference particleboards made from mature wood. However, the dimensional stability of manufactured boards was significantly reduced. Therefore, it can be concluded that the existing literature presents varying results regarding the impact of juvenile wood on the properties of wood-based panels, which justifies the need for further research to explain this effect. Moreover, despite the general trend toward producing low-density particleboards, which can be used both in single-layer form and in the production of sandwich composites, no research has been conducted on the use of juvenile wood for their manufacturing process.

This study aims to explore the potential of pine and birch juvenile wood as alternative raw materials in the production of low-density particleboards and to investigate their impact on both physical and mechanical properties of the boards.

## 2. Materials and Methods

**Figure 1:**
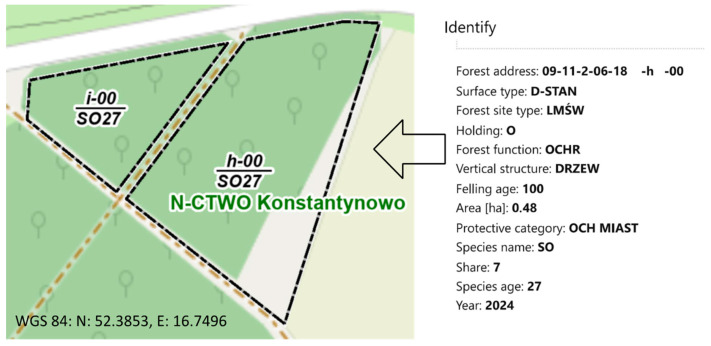
The area from which the materials for particleboard production were collected [38].

For the production of experimental particleboards, branchwood from juvenile Scots pine (*Pinus sylvestris* L.) and silver birch (*Betula pendula* Roth) was used. The pine wood was harvested from the Regional Directorate of State Forests near Poznań, Poland. It was obtained during the early thinning operations in a 25-year-old stand (Figure 1). This material was collected approx. two weeks following the completion of the thinning procedure. Birch wood was obtained from agricultural wastelands close to Poznań, Poland, where natural regeneration had occurred through self-seeding. The research material included branches and tops of pine and birch, with diameters of up to 7 cm. The number of annual rings ranged from 5 to 13, ensuring the material consisted entirely of juvenile wood for the study. Density of wood was determined according to Polish standard PN-77-D-04101 [37].

**Figure 2:**
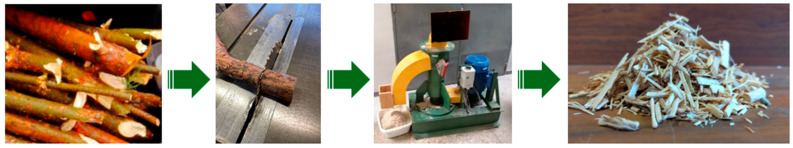
Preparation of wood particles for particleboard production.

The obtained biomass was initially cut into smaller pieces, approx. 4–5 cm in length, and ground into particles using a laboratory knife mill (Research & Development Centre for Wood-Based Panels, Czarna Woda, Poland). The resulting pine particles (PNE-JW) and birch particles (BIR-JW) were then sieved to remove larger fractions and dust. Next, wood particles were dried to a target moisture content of 3 ± 0.5% in a laboratory dryer with forced convection (Binder GmbH, Tuttlingen, Germany). A particle preparation process is shown in Figure 2.

Industrial pine particles (PNE-IP) obtained from mature wood were used as the reference material. These particles are intended for the production of the middle layer of particleboards at a local industrial plant.

**Figure 3:**
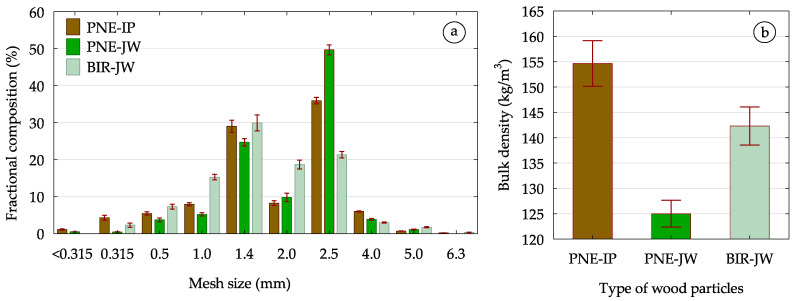
Characteristics of wood particles depending on the species and age of wood: (**a**) fractional composition, (**b**) bulk density.

To initially characterize the raw materials used in the study, their fractional composition was determined through sieve analysis and bulk density measurements (Figure 3). For the dominant fractions (1.4 mm and 2.5 mm), additional dimensional analysis was conducted, from which the degree of slenderness (λs), flatness (ψ), and width coefficient (m) of the particles were calculated using the equations previously used by Dukarska et al. [39,40] (Table 1).

The prepared particles were bonded using an industrial urea-formaldehyde (UF) resin, commonly employed in the production of wood-based panels for interior applications using a low-speed laboratory gluing machine. The resin, obtained from the Silekol (Kędzierzyn-Koźle, Poland), was characterized by a viscosity of 564 mPa·s, a density of 1.281 g/cm^3^, a solids content of 65.4%, a pH of 8.43, and a gel time at 100 °C of 58 s. To facilitate curing, 2% of a 20% ammonium nitrate solution (Chempur, Piekary Śląskie, Poland) was introduced as a hardener.

The following parameters were used in the production of the experimental particleboards: targeted board density of 550 kg/m^3^, dimensions of 380 mm × 670 mm × 19.5 mm, gluing degree of 8%, pressing temperature of 180 °C, unit pressure of 2.5 N/mm^2^, and pressing time of 18 s/mm of the final board thickness. The process of gluing the wood particles was carried out in a slow-speed gluing machine equipped with the LFG-5 pneumatic system (Devilbiss, Warsaw, Poland). In turn, a semi-automatic ATR 95 laboratory press (G. Siempelkamp GmbH & Co. KG, Krefeld, Germany) was used to press the boards. Two single-layer particleboards were produced for each type of material.

The produced particleboards were conditioned for 7 days at a temperature of 21 ± 2 °C and a relative humidity of 65 ± 2%. The suitability of juvenile wood for the production of low-density particleboards was evaluated by testing their mechanical properties, including internal bond (IB) according to EN 319 [41], bending strength (MOR), and modulus of elasticity (MOE) according to EN 310 [42], as well as their resistance to short-term water exposure, determined by measuring thickness swelling (TS) after 2 h of soaking in water based on EN 317 [43]. Each property was investigated using 10 samples.

The results obtained from the study were analyzed using Statistica 13.3 software with the use of one-way analysis of variance (ANOVA). Post hoc comparisons were performed using the Tukey’s HSD test. A *p*-value of <0.05 was considered statistically significant.

## 3. Results and Discussion

Particleboards, unlike wood-based boards made from solid wood or veneer, are characterized by a U-shaped density profile, with higher density in the surface layers and lower density in the core layer. The shape of the density profile significantly influences the properties of particleboards [44]. In the case of particleboards with a density profile where the outer layers exhibit a substantially higher density than the inner layer, the bending strength of the boards is usually enhanced [45]. On the other hand, when the density differences between the outer and inner layers are minimal, the internal bond reaches higher values, while bending strength decreases due to the lower capacity of the less dense outer layers to withstand higher stresses [44]. An important indicator of an optimal density profile is the ratio of the minimum core layer density to the average board density [46].

**Figure 4:**
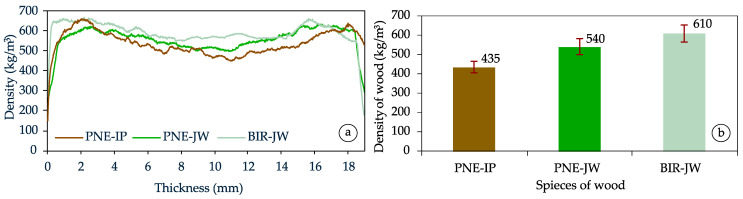
The outcomes of density measurements: (**a**) density profile of particleboards; (**b**) average density of wood.

Density profile measurements conducted on the manufactured particleboards revealed a distinct variation depending on the type of wood particles used. As shown in Figure 4, particleboards made from industrial particles demonstrated the most pronounced variation in density profile. These boards are characterized by a clear density peak in the outer layers (approx. 650 kg/m^3^), which occurs at a depth of up to 2 mm, with a significant decrease in density in the core layer. This phenomenon is attributed, in part, to the higher density and stiffness of the particles, as well as the greater structural stability of mature wood, which better transfers loads during the pressing process compared to juvenile wood.

The density profile of particleboards made from juvenile pine wood exhibited less noticeable difference between the outer and inner layers. The density peak occurred at a slightly greater depth from the surface, suggesting a more uniform distribution of density. This distribution is a result of the lower stiffness and greater susceptibility of juvenile wood particles to deformation during the pressing process. This observation is consistent with previous studies, which have shown that the reduced density of juvenile wood, relatively small number of latewood cells, higher proportion of thin-walled cells, changes in the microfibril angle, and shorter tracheids contribute to its lower strength and stiffness compared to mature wood [29,47,48,49,50,51,52].

When analyzing the density profile of boards made from juvenile birch wood, which showed the highest density among the tested raw materials, it was observed that the difference in density between the surface layers and the core layer was relatively small. This indicates that the pressing process did not result in a significant densification of the outer layers. These findings are consistent with the outcomes presented by Beck et al. [53], who also reported a less pronounced differentiation in the density profile of birch wood boards, with only a slightly higher density in the surface layers. According to Pipíška et al. [34], for higher-density wood species, such as birch, beech, hornbeam, oak, or willow, there is usually much less variety in a course of density profile. This phenomenon may be attributed to the challenges of achieving significant densification under high pressure and temperature. Lower-density wood is more susceptible to densification, particularly in the surface layers, compared to the core layer. Additionally, as demonstrated by Zhuang et al. [54] on the basis of the outcomes of the study on OSB, the difference in density between the surface and core layers of boards decreases as the density of the wood increases.

**Figure 5:**
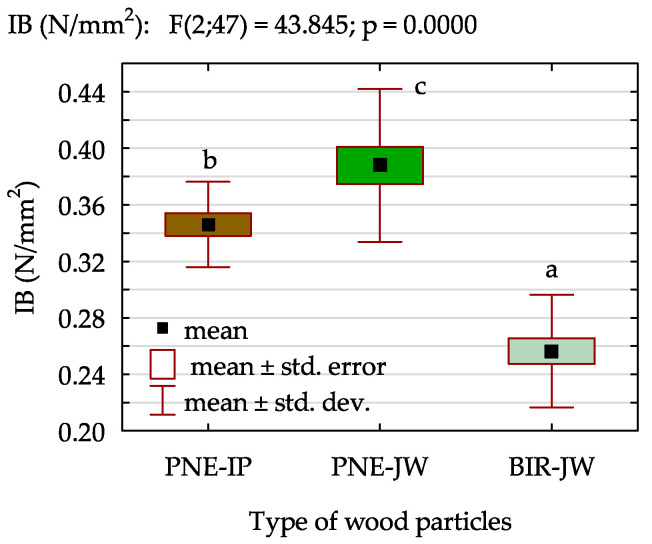
Internal bond of particleboards (a, b, c—homogeneous groups of mean values determined by one-factor ANOVA with Tukey’s test).

The differences observed in the density profile of the boards reflect the influence of the structural and density variations in the wood itself, as well as the bulk density of the particles. These factors significantly impact the mechanical properties of the resultant particleboards. As shown in Figure 5, boards made from pine particles, particularly those derived from juvenile pine (PNE-JW), exhibited higher internal bond (IB). Although the IB values for boards made from PNE-JW were only 8% higher than those made from industrial pine particles (PNE-IP), post hoc analysis revealed a statistically significant difference between the tested variants despite the relatively small differences in mean values. Boards produced from juvenile birch particles (BIR-JW) demonstrated the lowest mean IB value. Compared to the reference board, the IB value for this variant was approx. 25% lower and it was 32% lower compared to the board made from PNE-JW. However, despite the reduced density of the manufactured particleboards (550 kg/m^3^, compared to the typical density of 750 kg/m^3^ for traditional boards), all tested variants met the requirements specified in the EN 312 [55] for general-purpose boards intended for use in dry conditions (P1 boards).

Such a high internal bond of PNE-JW boards can be attributed to their greater elasticity and higher susceptibility to deformation. These properties enhance the contact area between glued particles and reduce the adhesive bond line thickness, which generally improves adhesion. Additionally, the increase in the density of mature wood tends to lower the IB value [34]. It is well known that the high-density wood is more challenging to bond due to its thicker cell walls and smaller pore diameters, which limit the adhesive penetration into the wood tissue. This observation aligns with Figure 4b, which illustrates the density of the wood used in the study. Furthermore, high-density wood species exhibit greater strength, allowing higher loads to be transferred to the bond line [56]. Another factor that may hinder optimal adhesive bonding is the higher content of extractives in juvenile wood compared to mature wood [57,58,59]. In the case of juvenile birch, extractives are more evenly distributed throughout the wood than in pine; however, their elevated concentration may create a barrier that hinders the adhesive penetration, which consequently negatively affects bonding quality.

**Figure 6:**
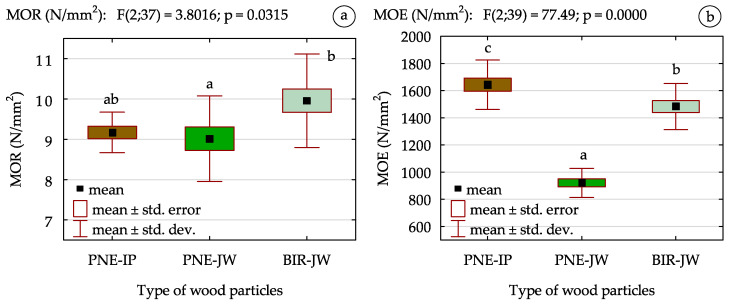
The results of (**a**) bending strength (MOR); (**b**) modulus of elasticity (MOE) of particleboard (a, b, c—homogeneous groups of mean values determined by one-factor ANOVA with Tukey’s test).

The bending strength (MOR) of the manufactured boards exhibited a different trend. As shown in Figure 6a, particleboards produced from pine wood, both juvenile and mature, demonstrated slightly lower MOR values (approx. 9% lower) compared to those made from BIR-JW. It is also important to notice that the obtained MOR values were only slightly below the requirements specified in the EN 312 [55] for P1-type boards with a thickness ranging from 13 to 20 mm (minimum required value is 10 N/mm^2^). Despite the reduction in board density, these results can be considered satisfactory, suggesting that their mechanical properties remain at an acceptable level. Therefore, it can be concluded that these boards have the potential to be used as core layers in multilayer panels, which are widely utilized in furniture manufacturing and construction applications, such as in door and wall structures.

The lack of statistically significant differences between particleboards made from mature and juvenile pine determined based on the analysis of homogeneous groups may be attributed to the findings of Tomczak and Jelonek [29]. The outcomes of their research showed that while mature wood exhibits slightly higher both static bending strength and strength quality factor at static bending, the differences are minimal, reaching up to 0.2% and 1.4%, respectively. Furthermore, in this study, single-layer boards of similar density were produced, and identical pressing conditions were applied across all tested variants. The standardized production parameters, including uniform pressing conditions and the use of particles with comparable sizes and shapes (especially in the case of PN-IP and PN-JW), most likely minimized the influence of wood age and species on bending performance. Markedly, Pipíška et al. [34] reported similar findings, showing no significant differences in MOR values among OSB produced from various types of juvenile wood. Furthermore, it seems that higher density of birch wood combined with the greater slenderness of BIR-JW particles contributed to the higher bending strength of manufactured particleboards. As previously noted, increased slenderness of particles positively influences MOR and MOE; however, as previously mentioned, it may also contribute to a reduction in IB [60,61].

Based on the outcomes presented in Figure 6b, it was found that the use of juvenile pine and birch wood instead of traditional raw materials significantly affected the modulus of elasticity (MOE) of the manufactured particleboards. The results of the post hoc test confirmed statistically significant differences between the tested variants. Overall, boards made from juvenile wood demonstrated lower MOE values compared to those produced from industrial particles. The most noticeable difference was observed in the case of using pine. Particleboards made from PN-JW had an MOE approx. 40% lower than those made from PN-IP. In contrast, the reduction in MOE for boards made from juvenile birch wood was only about 6%. This reduction in MOE observed for particleboards made from juvenile wood, particularly pine, probably resulted from differences in the structural characteristics between juvenile and mature wood. The annual rings in conifers, particularly those formed in the inner part of the cross section, are characterized by different properties compared to wood formed at a later growth stages [29]. According to Jelonek et al. [62], as the distance from the core of the trunk increases (increased proportion of juvenile wood), the resistance of wood to mechanical loads decreases and its elasticity increases. Additionally, mature wood may contain a higher content of reaction wood and increased number of knots, which can further influence its mechanical properties. The process of xylogenesis, determining wood structure and properties, is influenced by internal and external stress factors, including the weight of the tree crown, the overall mass of the tree, and environmental factors, such as wind [29,63,64,65]. It is important to note that variability in wood properties is an inherent characteristic of coniferous species, with the heterogeneity depending on the species. Juvenile wood is usually characterized by higher variability compared to mature wood. It was previously found that, depending on the sampling location, juvenile wood may present different mechanical parameters, yet it consistently demonstrates high elasticity [66]. For these reasons, the MOE of particleboards manufactured from juvenile wood is lower than that of boards made from mature wood.

Considering the potential application of tested particleboards in the production of interior-grade products, their thickness swelling (TS) and water absorption (WA) were additionally evaluated after a short-term, two-hour water immersion. These tests allow the evaluation of the dimensional stability under short-term water exposure and may be used as a complementary component of the analysis aimed at understanding their behavior under varying service conditions.

**Figure 7:**
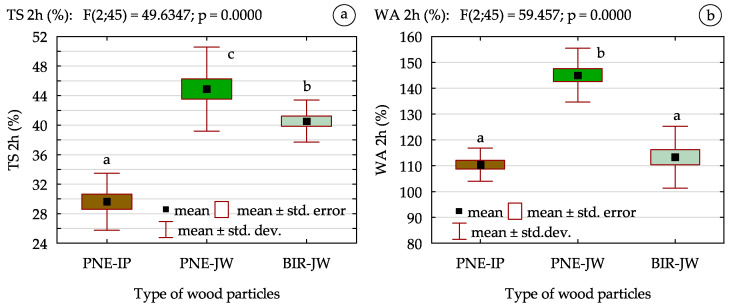
Water resistance of particleboards: (**a**) thickness swelling; (**b**) water absorption (a, b, c—homogeneous groups of mean values determined by one-factor ANOVA with Tukey’s test).

**Figure 8:**
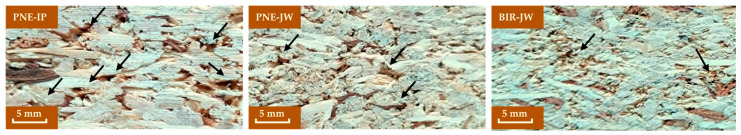
Cross-sections of particleboards (arrows indicate voids in the board structure).

The analysis of obtained results (Figure 7) indicates that boards made from juvenile wood exhibited higher values of both TS and WA compared to those made from industrial particles, which indicate lower water resistance. The most noticeable differences were observed in TS, where juvenile wood-based boards showed an increase of approx. 52% for PN-JW and 37% for BIR-JW compared to the reference particleboard. In terms of water absorption, a statistically significant increase of approx. 31% was observed only for boards made from juvenile pine. These findings indicate lower hydrophilicity of mature wood than juvenile wood, which is an important factor in particleboard production. The lower water resistance of particleboards made from juvenile wood particles, especially observed in the case of pine, may be attributed to differences in anatomical structure and physical properties. As previously mentioned, juvenile wood contains a higher proportion of thin-walled cells, a greater number of pores, and a higher earlywood-to-latewood ratio. These characteristics, along with its lower density, contribute to the greater hygroscopicity of the wood. Although birch wood is naturally denser than mature pine, in the case of juvenile wood it demonstrates higher porosity due to a greater number of void spaces, which facilitates more intense water absorption. On the contrary, mature pine has a more compact structure, thicker cell walls, and a lower earlywood content, which limits water penetration and reduces susceptibility to swelling. Additionally, mature wood is considered more dimensionally stable than juvenile wood [67,68]. The increased stability results from the natural processes, during which most cells reach their final dimensions and structural properties. Juvenile wood, in contrast, has a greater microfibril angle relative to the longitudinal axis of the cell, making it more susceptible to dimensional changes and deformation in axial direction under exposure to moisture conditions [69]. In mature wood, where microfibrils are more aligned parallel to the longitudinal axis, swelling in is usually lower. Moreover, as illustrated in Figure 8, the structure of the manufactured boards was also affected by the raw material used. Particleboards made from juvenile wood showed a more compact cross-section. This can be related to several factors, including the lower bulk density of this type of particles, their greater flatness and slenderness, and reduced stiffness. These characteristics facilitate the compression of the particles during pressing, resulting in a more uniform structure. However, the compact structure of boards made from juvenile wood also contributes to greater swelling. Due to the close arrangement of the particles, water penetrates directly into the wood, leading to increased swelling when exposed to moisture. In contrast, industrial particles from mature wood are thicker and more rigid, making their compression more challenging. Consequently, more voids are formed, which provide space for water without causing significant swelling of the wood, especially under short-term water exposure conditions.

Determining a direct effect of the chemical composition of raw materials on the dimensional stability of the manufactured wood-based materials is challenging. According to the literature, both juvenile and mature wood exhibit significant variability in chemical composition, influenced by factors such as tree species, climatic conditions, soil quality, forest habitat type, and growth conditions [58,70]. However, many studies indicate that juvenile wood shows higher content of water-soluble extractives, both in cold and hot water, compared to mature wood [57,58,71]. This characteristic, combined with the type of adhesive used, may have contributed to the reduced water resistance of particleboards manufactured from juvenile wood. Furthermore, UF resin itself is susceptible to hydrolysis under conditions of increased humidity or direct water exposure. It leads to the cleavage of chemical bonds within the adhesive joint. Even after a short soaking period, in this case two hours, the degradation of adhesive bonds may begin, reducing their capacity to maintain the particleboard’s structural integrity. Consequently, water penetrates more easily, which leads to greater swelling and water absorption [72].

## 4. Conclusions

This study compared the mechanical and physical properties of particleboards made from juvenile pine and birch wood with those from industrial particles of mature pine. The outcomes indicate that juvenile pine particleboards exhibited higher internal bond than those made of juvenile birch. The bending strength of both juvenile pine and juvenile birch boards was comparable or better than reference boards; however, juvenile pine boards had a 40% lower modulus of elasticity, which indicates significantly reduced stiffness. Furthermore, they showed higher short-term swelling and water absorption, potentially limiting durability in humid conditions. Juvenile birch particleboards had a 32% lower internal bond than those from industrial particles but maintained comparable bending strength, making them suitable for applications requiring good flexural properties. Their modulus of elasticity was only slightly reduced, while swelling increased by 37% and water absorption remained similar to other variants. Overall, juvenile birch particleboards emerged as the most promising option, offering good bending strength, satisfactory modulus of elasticity, and relatively good water resistance compared to juvenile pine. Despite their lower density, these boards show potential for applications such as, for example, production of sandwich panels. Overall, despite some flexural strength limitations that still exist, they meet the requirements for P1 board in terms of the internal bond, which shows the potential for further research on their industrial viability as a cost-effective alternative to traditional particleboards.

## Figures and Tables

**Table 1:** The outcomes of dimensional analysis of the particles used for particleboard manufacturing.

Species of Wood Particles	Fraction (mm)	Shape Factors of Wood Particles		
		Degree of Slenderness (λs)	Degree of Flatness (ψ)	Width Coefficient (m)
PNE-IP	1.4	12.42	1.02	12.16
	2.5	12.64	1.53	8.27
PNE-JW	1.4	17.11	2.12	8.10
	2.5	20.50	2.22	9.22
BIR-JW	1.4	16.78	2.18	7.69
	2.5	18.55	2.13	8.71

## Data Availability

The original contributions presented in the study are included in the article, further inquiries can be directed to the corresponding authors.
